# Potential Compounds as Inhibitors of Staphylococcal Virulence Factors Involved in the Development of Thrombosis

**DOI:** 10.3390/toxins17070340

**Published:** 2025-07-04

**Authors:** Anna Lichota, Krzysztof Gwozdzinski, Monika Sienkiewicz

**Affiliations:** 1 Department of Pharmaceutical Microbiology and Microbiological Diagnostics, Medical University of Lodz, 90-151 Lodz, Poland; monika.sienkiewicz@umed.lodz.pl; 2 Department of Oncobiology and Epigenetics, University of Lodz, 90-236 Lodz, Poland; krzysztof.gwozdzinski@biol.uni.lodz.pl

**Keywords:** staphylococcal virulence factors, therapeutic strategies, staphylococcal infections, thrombosis

## Abstract

For many years, staphylococci have been detected mainly in infections of the skin and soft tissues, organs, bone inflammations, and generalized infections. Thromboembolic diseases have also become a serious plague of our times, which, as it turns out, are closely related to the toxic effects of staphylococci. *Staphylococcus aureus,* because of the presence of many different kinds of virulence factors, is capable of manipulating the host’s innate and adaptive immune responses. These include toxins and cofactors that activate host zymogens and exoenzymes, as well as superantigens, which are highly inflammatory and cause leukocyte death. Coagulases and staphylokinases can control the host’s coagulation system. Nucleases and proteases inactivate various immune defense and surveillance proteins, including complement components, peptides and antibacterial proteins, and surface receptors that are important for leukocyte chemotaxis. On the other hand, secreted toxins and exoenzymes are proteins that disrupt the endothelial and epithelial barrier as a result of cell lysis and disintegration of linking proteins, which ultimately increases the risk of thromboembolism. In this review, we discuss various virulence factors and substances that may inhibit their activity.

## 1. Introduction

Overuse and misuse of antibiotics has dramatically increased bacterial resistance, which in turn has contributed to serious therapeutic problems. Treatment becomes ineffective, so combined therapy is very often needed, and all this brings about adverse consequences for the patient [1]. Therefore, virulence factors become a target in the fight against bacteria. We are looking for compounds that have a direct effect on bacterial virulence factors by reducing their production and neutralizing their activity. Perhaps, such actions will help avoid the spread of antibacterial drug resistance [2].

*Staphylococcus aureus* is known to be the most common species responsible for many human infectious diseases in the environment [2]. The *Staphylococcus* genus is characterized with a wide range of virulence factors which are responsible for the hemolysis of blood cells, coagulation of plasma, and production of many enzymes and extracellular toxins, which enables adhesion and invasion of host tissues. They do not only induce difficult-to-treat infections but also cause food contamination by producing thermostable enteric toxins. They pose a danger particularly as etiological factors of hospital infections due to acquisition of resistance mechanisms but also, very importantly, due to their particular resistance to antimicrobial agents and desiccation [3].

Typical staphylococcal infections include minor skin and soft tissue infections, such as abscesses, furuncles, and impetigo. However, generalized infections, such as bacteremia, sepsis, and toxic shock syndrome (TSS), are particularly dangerous to health and life. Furthermore, *S. aureus* is a major factor in hospital infections, including surgical site infections, and due to its ability to form bacterial biofilm, it poses an increased risk of infectious complications in procedures using biomaterials. Hence, complications after cardiac and orthopedic surgeries or in intensive care units are very common [4].

Due to the fact that staphylococcal virulence factors are responsible for disease pathogenesis, tissue injury, and treatment failure, they are becoming viable therapeutic targets. Treatments targeting virulence without direct toxicity to the inciting pathogen can support traditional antimicrobial treatment [5].

It is expected that a treatment which involves combating the virulence factors of *S. aureus* will inhibit the phenomenon of the selection of resistant strains and will have little impact on the host microbiota [6]. Bacteria contained in the microbiota have developed mechanisms for competing with *S. aureus*. The mechanisms include the production of antagonistic autoinducing peptides, release of antibacterial compounds, such as antibiotics and bacteriocins, and in response, *S. aureus* produces compounds that inhibit the development of microbiota [7].

According to data, *S. aureus* activates platelets via secreted proteins, such as α-hemolysin—Hla—or indirectly, involving host extracellular matrix proteins. Hla is a pore-forming toxin which shows activity in disrupting epithelial barriers, directly activates human platelets, and leads to their activation and aggregation. Direct Hla binding and clot destabilization has major medical implications. According to Jahn et al. (2022) [8], thrombus stabilization by the inhibition of Hla might reduce the risk of microthrombi dissemination.

*S. aureus* also produces the bicomponent pore-forming leukocidins LukSF, also referred to as Panton–Valentine leukocidin (PVL), LukED, and LukAB [9]. Exotoxins of *S. aureus,* for instance, PVL, are responsible for leukocyte lysis and injury to endothelial surfaces [10]. The literature states that staphylococcal infections, such as acute hematogenous osteomyelitis, pneumonia, or sepsis, which ended in death, were connected with deep venous thrombosis. Therefore, it is advisable in cases of diagnostic patients with infections caused by hypervirulent strains of staphylococci to evaluate for deep venous thrombosis in extremities and in unusual sites [11].

In this review, we discuss various virulence factors and substances that may inhibit their expression and activity. Large-scale scientific studies show the most important research areas: plant secondary metabolites, peptides with antibacterial activity, monoclonal antibodies, and nanoparticles. We have particularly focused on the antithrombotic activity of natural compounds.

## 2. Therapeutic Strategies in Neutralization of Virulence Factors

### 2.1. Natural Product

The emergence of multi-drug-resistant *S. aureus* strains increased the interest in new compounds of natural origin. Many substances, including compounds of plant origin, are natural inhibitors of the toxin released by staphylococci. The mechanism of action of these substances involves blocking the access of the toxins to cell membranes or inhibition of the formation of pores in the cell membranes of the host [12].

Natural compounds with anti-toxin properties or that target the agr-quorum sensing (QS) system may be helpful in anti-staphylococcal therapy. In literature reports, morin hydrate and oroxylin glycoside are toxin-neutralizing compounds, and isorhamnetin, chrysin, puerarin, naringenin, ω-hydroxyemodin, and ambuic acid are compounds directed at the agr-QS system [13].

Flavonoids can interact with three groups of proteins and enzymes which are involved in the growth and metabolism of bacteria. One group consists of flavonoids which act on enzymes involved in the metabolism of DNA and proteins. Bacterial topoisomerases, helicases, DNA gyrases, and ribosomes can be mentioned here. The second group includes flavonoids which affect proteins and membrane enzymes involved in cell transport, bioenergetics, maintenance of homeostasis, cell wall stability, and lipid metabolism. The third group consists of flavonoids, which have a beneficial impact on the pathogenesis of microorganisms and thus counteract the production of toxins and formation of biofilm [14].

**Figure 1:**
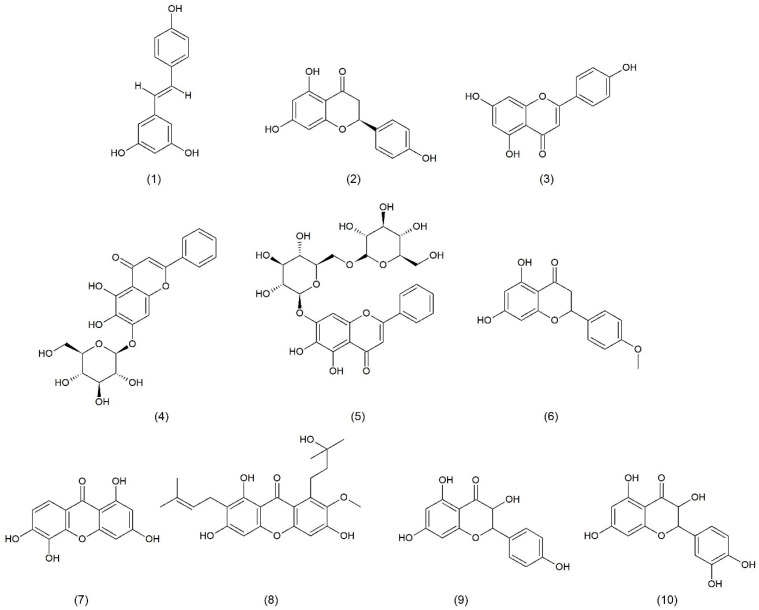
Chemical structure of resveratrol (**1**), naringenin (**2**), apigenin (**3**), oroxylin A (**4**), oroxin B (**5**), ponciretin (**6**), 1,3,6,7-tetrahydroxyxanthone (**7**), D-garcinon (**8**), kaempferol (**9**), and quercetin (**10**).

α-toxin may be a target for natural anti-infective drugs used in infections caused by *S. aureus*. Resveratrol (stilbene derivative) (Figure 1), being a natural compound found in blueberries, cranberries, mulberries, or grapes, is one of such medications. According to literature data, resveratrol used at a concentration of 1/8 MIC inhibits the hemolytic capacity of *S. aureus*. However, no inhibition of bacterial growth was observed. It was revealed that resveratrol downregulates the transcription of the Hla gene, encoding α-hemolysin (Hla), and reduces RNAIII transcription within the agr system. In addition, at subinhibitory concentrations, resveratrol significantly reduces the production of protease and lecithinase [15].

Flavonoid naringenin (Figure 1), mainly found in grapefruit but also in many other fruits and herbs, strongly decreases the amount of α-toxin in an *S. aureus* culture [16].

Apigenin (Figure 1), a flavonoid that is found in a variety of citrus fruits, herbs, and vegetables, such as grapefruit, tarragon, coriander, spearmint, and basil, as well as in onions, tomatoes, peas, and broccoli, inhibits the transcription of the Hla and agrA genes that encode Hla, which decreases the production of Hla in *S. aureus* and plays an anti-inflammatory role [17].

Oroxin A (Figure 1), oroxin B (Figure 1), and oroxylin A7-O-glucuronide, present in fruits like strawberries, apples, and grapes, prevents the conversion of the hemolysin monomer to oligomer [6].

Ponciretin (Figure 1), a flavonoid, inhibits the hemolytic and cytolytic activity of the γ-toxin [18]. Compounds contained in *Garcinia pedunculata* and 1,3,6,7-tetrahydroxyxanthone (Figure 1) dye extracted from mango leaves also demonstrate similar properties. Analyses of the docking revealed that some natural bioactive compounds, which are found in *G. pedunculata*, inhibit the activity of the γ-toxin of *S. aureus*. Two compounds that were active against the γ-toxin were identified: 1,3,6,7-tetrahydroxyxanthone and D-garcinon (Figure 1), with a high binding affinity to the target protein. These compounds are potent inhibitors that could be further used to develop new drugs for the treatment of staphylococcal infections [19].

Kaempferol (Figure 1) is also an effective antibacterial flavonoid. Direct inhibition of bacterial DNA gyrase, which was initially detected in *E. coli* [20], is an important antibacterial mechanism of action of kaempferol. Further studies showed that this flavonoid also effectively inhibits DNA gyrase in methicillin-resistant *S. aureus* (MRSA) [21]. Moreover, kaempferol inhibited DNA helicase, in particular SAPriA, in *S. aureus* [22]. It was also shown that kaempferol inhibited biofilm formation and reduced the activity of *S. aureus* sortase A (SrtA) as well as the expression of adhesion-related genes [23]. Chan et al. (2014) [24] showed that galloylated derivatives of kaempferol in the 2″,3″,4″ (tri-*O*-galloyl) positions and quercetin (Figure 1) in the 3″,4″ (di-*O*-galloyl) positions as well as quercetin in the 2″,3″,4″ (tri-*O*-galloyl) positions, isolated from *Calliandra tergemina* leaves, had a lytic effect on MRSA.

Apart from flavonoids, tannins also showed an anti-hemolytic activity against *S. aureus*. These compounds inhibited α-toxin, and their activity depended on the chemical structure and molecular mass. The tannins containing the valoneoyl group (T3 and T4) were less active than other compounds. Moreover, it was shown that the anti-hemolytic activity was related to the order of the lipid monolayer of the plasma membrane of red blood cells (RBCs) [2].

**Figure 2:**
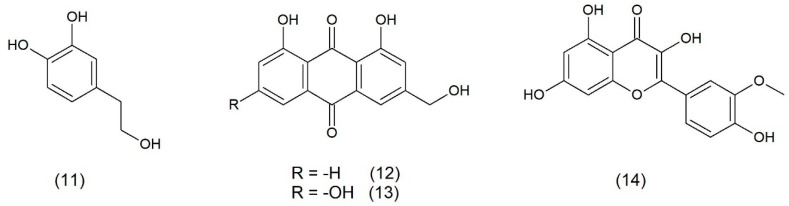
Chemical structure of 4-hydroxytyrosol (**11**), aloe-emodin (**12**), ω-hydroxyemodin (**13**), and isorhamnetin (**14**).

It was confirmed that 4-hydroxytyrosol (Figure 2), found in olive oil, reduced effects of the staphylococcal enterotoxin A (SEA) in in vitro conditions both as a pure substance as well as in a mixture with raw material components. Studies have shown that perilla oil obtained from *Perilla frutescecens* from the *Lamiaceae* family decreased the production of enterotoxins A and B and toxic shock syndrome toxin-1 (TSST-1) by *S. aureus* [6].

ω-hydroxyemodin (Figure 2) has appeared to be a compound acting through the agr system that controls the production of virulence factors necessary for *S. aureus* to induce invasive skin and soft tissue infections (SSTIs). A reduction in dermonecrosis during treatment with ω-hydroxyemodin was associated with the elimination of bacteria and a reduction in the transcription and expression of inflammatory cytokines at the site of infection. ω-hydroxoksyemodin enhanced the immune cell killing of *S. aureus* in vitro in an agr-dependent manner. It appears that inactivating the bacteria by inhibiting *S. aureus* QS may enhance the host’s innate immune response and reduce inflammation [25].

Inhibitory properties against the α-toxin were also demonstrated by aloe-emodin (Figure 2), which is a natural compound obtained from aloe. The hemolytic activity was manifested by an inhibited oligomerization of the α-toxin and binding of aloe-emodin to K110, T112, and M113 of the toxin. The aloe-emodin reduced damage to human lung epithelial cells and mouse lung macrophages induced by *S. aureus* [26].

Flavonoid isorhamnetin (Figure 2) is produced by common sea buckthorn (*Hippophae rhamnoides*) and gingko (*Ginkgo biloba*). Isorhamnetin downregulates the RNAIII gene encoding Hla and inhibiting Hla transcription [27].

**Figure 3:**
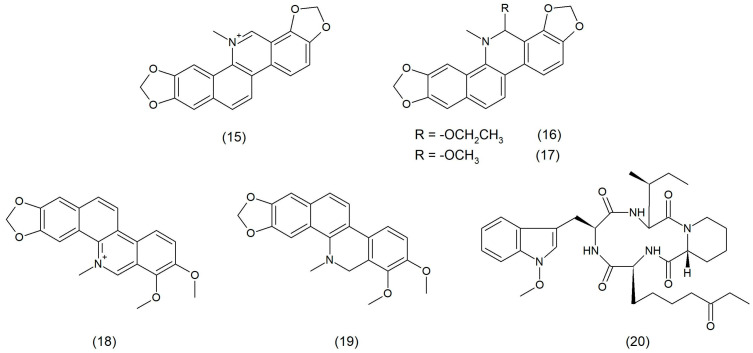
Chemical structure of sanguinarine (**15**), ethoxysanguinarine (**16**), 6-methoxydihydrosanguinarine (**17**), chelerythrine (**18**), dihydrochelerythrine (**19**), and apicidin (**20**).

The main compounds exhibiting antimicrobial, anti-inflammatory, and antioxidant properties are alkaloids from the *Macleaya cordata* species, such as sanguinarine (Figure 3), ethoxysanguinarine (6-ES) (Figure 3), 6-methoxydihydrosanguinarine (6-MS) (Figure 3), chelerythrine (CH) (Figure 3), and dihydrochelerythrine (DICH) (Figure 3). Alkaloid 6-ES was shown to kill MRSA rapidly, possibly due to interference with the membrane and metabolic functions, including reactive oxygen species (ROS) production. Moreover, 6-ES directly inhibited the hemolytic activity of Hla, reduced inflammatory responses, and reduced intracellular MRSA. 6-ES containing hydrogel was shown to promote wound healing in MRSA-infected mice. These results confirmed that 6-ES is a new potential candidate or lead compound with antibacterial, antiviral, and host-immunomodulating activity in the fight against bacterial infections [28].

Decreased effectiveness of antibiotics used clinically against drug-resistant *S. aureus* becomes a challenge in the search for other substances which are more effective in the fight against this pathogen. A fungus metabolite, called apicidin (a cyclic tetrapeptide) (Figure 3), which counteracts both resistance and virulence, is one of active substances against MRSA. Apicidin is a specific QS inhibitor which antagonizes the agr system in MRSA in a non-biocidal manner. This compound was shown to mediate the suppression of MRSA pathogenesis by detecting QS at sites of infection in vivo. In addition, apicidin attenuates MRSA infection by promoting the host defense, which is associated with the enhancement of innate effector responses, particularly through an increased accumulation of neutrophils at sites of infection [29].

**Figure 4:**
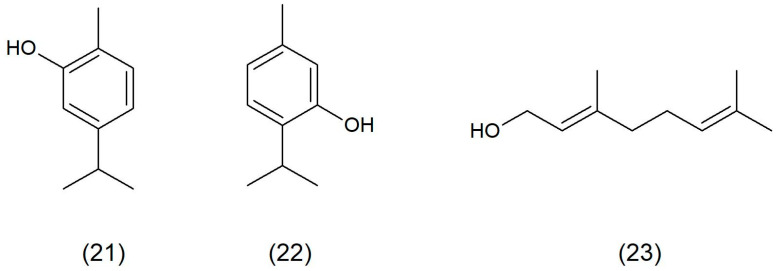
Chemical structure of carvacrol (**21**), thymol (**22**), and geraniol (**23**).

Research data reveal that phenolic compounds, such as carvacrol (Figure 4) and thymol (Figure 4), at sublethal concentrations, can inhibit the hemolytic and nuclease activities of *S. aureus*. These compounds are responsible for changes in the cell membrane of staphylococci, which affects the production of these virulence factors. In turn, staphylococci need nucleases to oppose the host’s neutrophils, as, without them, they are unable to defend themselves against the host’s immune system. These phenolic compounds were also found to inhibit the pore formation, which also promotes the defense mechanisms of the immune system. The sublethal treatment of carvacrol and thymol also effectively decreases the biofilm-formation ability of *S. aureus,* which is probably caused by reduced cell surface hydrophobicity [30]. Hayati et al. (2020) [31] showed the ability of carvacrol to inhibit the production of *S. aureus* toxins ent A and ent B.

According to Cui et al. (2019) [32], oregano essential oil contains mainly carvacrol. The investigation found that this compound can reduce the activity of the PVL toxin by almost 65% and can form chimeras with DNA.

Hao et al. (2021) [33] reported that the oregano essential oil causes damage to the cell membrane, leading to changes in its permeability, and also inhibits respiratory metabolism.

The effect of carvacrol on the transport of substances through the plasma membrane is to limit the secretion of bacterial toxins [34]. By disrupting the proton gradient, it affects the synthesis of adenosine triphosphate (ATP) and inhibits efflux pumps, which leads to the production of ROS and weakens the activity of virulence factors, i.e., SE and coagulase. It also has a destructive effect on QS by inhibiting the autoinducer-2 (AI-2) system and the formation of the *S. aureus* biofilm [35].

Geraniol (Figure 4) is an acyclic isoprenoid monoterpene that is produced by many aromatic plants, such as roses, geraniums, and lemons. It is used as an ingredient of essential oils in cosmetics, medicine, and aromatherapy. Moreover, geraniol has various biological activities, including anti-inflammatory, antioxidant, anticancer, antibacterial, hepatoprotective, cardioprotective, and neuroprotective effects [36]. It was shown that geraniol is not only characterized with anti-inflammatory and antioxidant properties but also protects mice in vivo against systemic MRSA infection [37]. The antibiofilm activity involving the inhibition of MRSA and MSSA strains’ biofilm formation and elimination shows that also geraniol decreases polysaccharide intercellular adhesin (PIA) production [38]. Geraniol also releases eDNA and is responsible for autolysis by inhibiting the gene expression of sarA and atlA. Morever, geraniol can improve the sensitivity of staphylococci on H_2_O_2_ and downregulate crtM gene expression, which is responsible for the production of staphyloxanthin, favoring the survival of the bacteria. Peng et al. (2023) [34] showed that carvacrol at sub-inhibitory concentrations is able to inhibit the expression of sarA and agrA. Inhibited expression of icaA, icaB, icaC, sarA, and agrA genes reduces the production of PIA, which prevents adhesion and destabilizes the biofilm.

### 2.2. Antimicrobial Peptides

Antimicrobial peptides (AMPs), substances produced by organisms, facilitate the survival of those organisms and enable them to colonize living environments [39]. According to recent studies, AMPs and small molecules can penetrate the cell membrane of *S. aureus*, inhibit phospholipid biosynthesis, or inhibit the passage between the periplasm and the exterior of the cell. AMPs and small molecules can also destroy the bacterial biofilm and downregulate the virulence genes of *S. aureus* [40].

**Figure 5:**
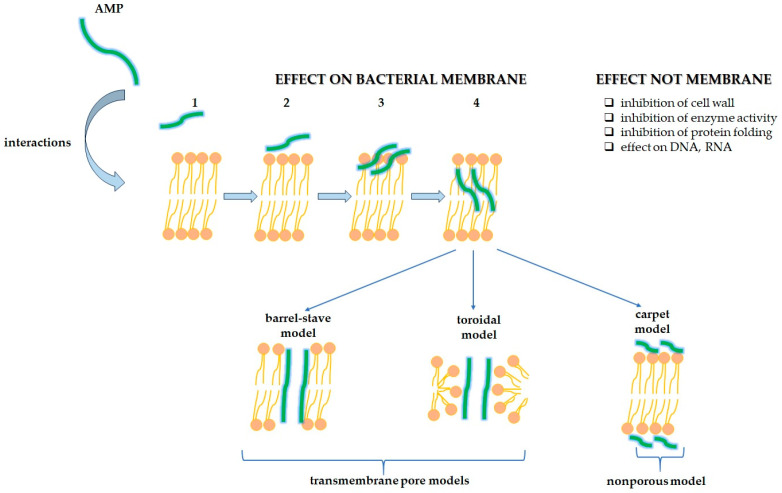
Different models of the antibacterial mechanism of AMP action. The basis of these models is the interaction of AMP with negatively charged bacterial membranes, leading to increased permeability of the cell membrane, and its lysis, which ultimately leads to cell death.

As shown in Figure 5, AMPs have different mechanisms of killing bacteria. AMPs mainly interfere with the cytoplasmic membrane and may carry out inhibition of DNA, RNA, and protein synthesis; inhibition of enzyme activity and cell wall synthesis; and stimulation of the release of lyase contributing to the damage of cell structures. AMPs bind to bacterial membranes through three models: barrel-stave, toroidal pore, and carpet. The barrel-stave model is a process in which AMPs are vertically positioned between the membrane, forming an ion channel. Pores allow for the leakage of cellular contents. In the toroidal pore model, pores are generated by both AMPs and the phospholipid head groups. Lipids and AMPs collaboratively form transmembrane channels. Meanwhile, in the carpet model, the AMPs arrange themselves in a parallel fashion of the cell membrane, and after reaching a critical threshold, they destroy the membrane structure [41,42,43,44].

There is also a model of a sinking raft, which explains how AMPs, forming peptide trimers, are incorporated into the outer lipid monolayer and then translocated to the inner lipid monolayer of the membrane [40].

The Food and Drug Administration (FDA) approved the following AMPs for clinical use: gramicidin, colistin, polymyxin B, daptomycin, vancomycin, oritavancin, dalbavancin, and telavancin. What is more, these substances are considered “last line” antimicrobials and are active even against multidrug-resistant (MDR) bacteria [45].

AMPs belong to five families: linear α-helix peptides, β-sheet peptides, peptides with both α-helix and β-sheet, peptides without α-helix or β-sheet peptides, and topologically complex AMPs [46]. α-defensins are divided into human neutrophil peptides (HNP1-4) and human α-defensin 5 and 6 (HD5 and HD6). α- and β-defensins, cathelicidin LL-37, and histatins can be found in humans [47].

Production of hemolysins and superantigens required for *S. aureus* infection is inhibited by human neutrophil α-defensin-1 (HNP-1), yet human β-defensin 1 (HBD-1) inhibits only hemolysin [48]. Virulence factors can be attached to the cell wall by special enzymes, as it happens in Gram-positive bacteria, which produce sortase enzymes, located on one or both sides of the cell membrane. Peptides can affect the proteins necessary for secretion and virulence factor assembly, as polymyxin B and HNP-1 at sub-inhibitory concentrations can bind to anionic lipids of the so-called ExPortal, which affects the secretion of both cysteine protease and cytolysin. Small dipeptides, one of which can be cyclo (L-Phe-L-Pro) dipeptide, are produced by Lactobacilli. They inhibit the growth of bacteria, fungi, and viruses and also suppress the formation of bacterial exotoxins by interfering with cognate QS-autoinducers. Cyclo (L-Phe-L-Pro) dipeptide suppresses the production of staphylococcal exotoxins (TSST-1) by affecting the agr QS-system [49].

Cathelicidins exhibit multidirectional protective immunomodulatory action by inducing chemotaxis. They can disintegrate microbial cell membranes by creating pores. LL-37 human cathelicidin is such an example. It can probably form an amphipathic α-helix by penetrating through the membrane and creating holes in it [50]. Molecular docking studies on human cathelicidin (LL-37) and its segments with LukS and LukF subunits of PVL toxin showed 34 molecular interactions of LL-37 with the LukS subunit of the PVL toxin. LL-37 may be a potential inhibitor of MRSA virulent toxins that may be used to treat nosocomial infections in healthcare settings [51,52].

It was found that a host defense peptide, i.e., fowlicidin-1, stimulates innate and adaptive immunity. In an animal model, its analogs protect mice from MRSA infection [46].

Bacterial RNA synthesis, mainly transcription, being a new target for antimicrobial agents, is also a subject of research. The interaction between NusB and NusE proteins is reported to be associated with bacterial cell viability. Nusbiarylins are one of compounds targeting NusB and NusE transcription factors [53]. Nusbiarylin-MC4 and its derivatives inhibit the growth, cellular respiration, and transcription. They also reduce the activity of virulence factors, such as staphylococcal α-toxin and PVL [54].

### 2.3. Monoclonal Antibodies and Vaccines

Immunotherapy is a very valuable tool in the fight against the spread of infectious diseases. New substances which could stimulate a strong humoral and cellular response are being sought [55]. In animal model studies, it was possible to develop anti-toxin antibodies that neutralize single virulence factors [13].

Due to the increasing resistance of *S. aureus* to commonly administered antimicrobial drugs, monoclonal antibodies, including those directed against virulence factors of *S. aureus*, may be an alternative treatment option. The drugs might include KBSA301 or MEDI4893, being full human IgG1 antibodies that specifically neutralize Hla and protect host cells [56].

Studies have shown that in immunocompromised mice with *S. auerus* pneumonia, passive immunization with monoclonal antibody MEDI4893* reduced the number of bacteria and influenced the survival rate. Moreover, significantly better results of treatment with vancomycin or linezolid in combination with this antibody were noted. This indicates that MAb-mediated neutralization of AT might be potentially administered in prevention and adjunctive therapy in immunocompromised patients [57].

Results of animal tests showed that the monoclonal antibody YG1 prevents binding to A549 cells and blocks the Hla-mediated lysis of rabbit erythrocytes. YG1 is also able to protect against peritoneal infection, bacteremia, and pneumonia in mice. This indicates that YG1 may be applied and serve as a promising protective strategy against *S. aureus* infection [56].

From the point of view of effective protection, the effect of inhibiting the activity of several toxins by a monoclonal antibody at the same time is desirable. Kailasan et al. (2022) [58] described that ASN-1 mAbs can neutralize Hla and four leukocidins (LukSF-PV, LukED, HlgAB, and HlgCB), and ASN-2 inactivates the fifth leucocidin, LukGH. ASN100, being a combination of ASN-1 and ASN-2, exhibits protective activity towards granulocytes, monocytes, NK-cells, and T-lymphocytes. The inhibition of toxins by ASN100 may block the cytolytic activity of *S. aureus* towards human target cells in vitro [58,59].

Bicomponent leukocidins γ-hemolysins (HIgAB and HIgCB), PVL, and LukED and LukAB are involved in evading the host immune response by lysing phagocytic cells. Research has shown that intravenous immunoglobulin (IVIg) used in clinical practice can neutralize not only *S. aureus* toxins, such as PVL, hemolysin, and TSST-1, but also LukAB [60]. An optimal dose of the drug should be adjusted individually, taking into account the number of neutralizing antibodies [61].

M0313, an anti-SEB human monoclonal antibody, inhibited the SEB activity, inducing a mouse splenic lymphocyte and human peripheral blood mononuclear cell proliferation and cytokine production. Mice treated with SEB-expressing bacteria survived much more often. The neutralization capacity of M0313 was related to SEB binding, blocking the major histocompatibility complex II and T-cell receptor [62].

Research conducted by Aguilar et al. (2017) [63] proved that MAb-4G3 (IgG2b), mAb-5G2 (IgG1), and mAb-9H2 (IgG1) inhibit SEK-induced proliferation and cytokine production of human immune cells.

In studies on a mouse model of *S. auerus* pneumonia, a new protective strategy against the hemolytic activity of the bacteria was developed. Anti-LukF mAbs, SA02 and SA131, demonstrated a specific neutralization activity to LukSF-PV, whereas SA185 exhibited cross-neutralization to LukSF-PV, γ-hemolysin HlgAB, and leukotoxin ED [58].

A comprehensive protective effect is demonstrated by monoclonal antibodies targeting several virulence factors. Hla-F#5 is an anti-toxin that neutralizes Hla and bi-component leukocidins, anti-PVL mAbs are for PVL and γ-hemolysin, and anti-LukS-mut9 is for PVL and other leukotoxins. In contrast to huMAb-154, mAb 20B1 and soluble Vβ protein are SEB-neutralizing anti-toxins [13].

Despite there being many natural and synthetic compounds described above, all vaccination attempts to prevent infection with virulent strains of *S. aureus* in humans have been unsuccessful. This applies to vaccines that would produce high titers of opsonic antibodies to *S. aureus* surface antigens. It is known that staphylococcal toxins, such as pore-forming toxins and superantigens, constitute an important group of virulence factors. The application of antibodies to these toxins would provide therapeutic benefits, as opposed to previous vaccination trials [64].

Pore formation in cell plasma membranes is one of major factors of MRSA virulence in the murine model of *S. aureus* pneumonia. It was shown that the level of α-toxin expression by *S. aureus* strains is directly related to their virulence. Active immunization with the modified α-toxin (Hla_H35L_) leads to an antigen-specific IgG response and protects against staphylococcal pneumonia. In addition, the transfer of the α-toxin-specific antibodies protects the animals from *S. aureus* infection and prevents damage to human lung epithelial cells during the infection. It appears that vaccination or α-toxin immunotherapy may be effective in preventing *S. aureus* pneumonia in humans [65].

*S. aureus* bacteria is special because of its multiple virulence factors. This poses a serious obstacle to the development of an effective vaccine stimulus, which would trigger to create functional antibodies capable of combating defense mechanisms of the pathogen [66]. However, there are reports on the development of new vaccines that stimulate the immune system to respond humorally as well as cellularly against staphylococcal toxins.

It was found that Hla_HRE_ immunization contributes not only to Hla neutralization, but also, as studies have shown, to enhancement and protection of antigen-specific T responses to *S. aureus* infection. Moreover, an immunized mother passes on protection to her child [67].

Adhikari et al. (2015) [68] described that polyclonal antibody generated by attenuated mutant of LukS-PV (PVL-S subunit)—LukS-mut9 can neutralize leukotoxic and hemolytic bicomponent toxins. They also proved that the risk of sepsis in patients with a higher antibody titer against those toxins is significantly lower.

Positive results manifesting with the production of neutralizing antibodies, directed against α-toxin and PVL toxins, were obtained after the administration of a vaccine recombinant α-toxoid (rAT) and a sub-unit of PVL (rLukS-PV) either alone or in combination [69].

It was also possible to obtain both a humoral response in the form of antibodies neutralizing toxins and a cellular response Th1/Th17 after application of a 4-component vaccine composed of mutants of S and F subunits of PVL (LukS_mut9_ and LukF_mut1_) and a double mutant of Hla (Hla_H35L/H48L_) [70].

IBT-V02 appeared to exhibit a broad spectrum of activity against extracellular staphylococcal toxins by forming pores. This multi-component toxoid vaccine neutralizes Hla, PVL, leukocidin AB (LukAB), and superantigens TSST-1, SEA, and SEB [71].

A safe, well-tolerated, and highly immunogenic recombinant detoxified vaccine against toxic shock syndrome toxin 1 variant vaccine (rTSST-1v) was also developed. Two doses of the vaccine are sufficient to produce a high-antibody titer, which provides protection against TSS and septic shock [72]. Schoergenhofer et al. (2024) [73] observed that cumulative doses of up to 300 μg were safe, well-tolerated, and highly immunogenic. Two doses of 100 μg rTSST-1v provided the most persistent immune response.

### 2.4. Nanoparticles as Alternative Therapeutic Compounds

Nanotechnology is another option for treating infections, including those of *S. aureus* etiology in the post-antibiotic era. Wide-scale studies are being conducted on liposomal and polymer drug carriers, as well as metal vectors, e.g., gold NPs. Gold nanoparticles are able to penetrate the cell membrane, destroy cellular structures, and stimulate the formation of ROS. Elimination and neutralization of staphylococcal toxins is particularly important in staphylococcal infections due to their devastating impact on the body, including the cardiovascular system [1].

**Figure 6:**
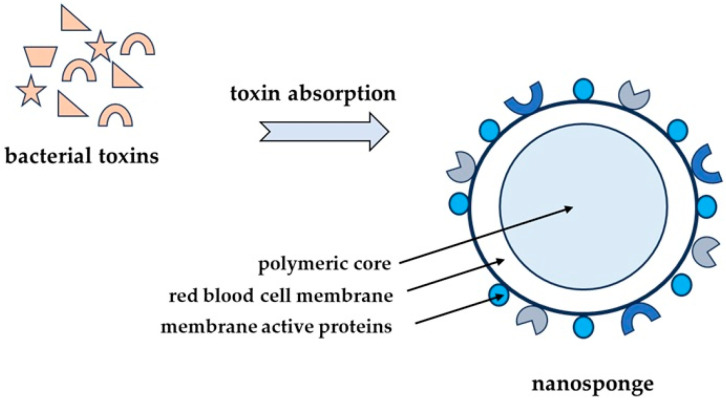
The nanosponges consist of a polymer core covered with bilayer membranes of human RBCs, which could absorb bacterial toxins, and they transfer them away from the cellular targets. The toxins bound by the nanosponge prevent the attack on the host cell targets, protecting them from damage.

Nanosponges, obtained by coating polylactic-co-glycolic acid (PLGA) polymer nanoparticles with membranes of RBCs, are an interesting solution among nanoparticles (Figure 6). Such modified nanoparticles serve as a decoy of toxins, binding and neutralizing many toxins with a hemolytic effect, regardless of their structure. Tests with model pore-forming toxins, such as melittin, MRSA α-toxin, *Listeria monocytogenes* listeriolysin O, and group A streptolysin O, showed that nanosponges are able to completely inhibit toxin-induced hemolysis in a concentration-dependent manner [74,75].

Therefore, the nanosponge may be a detoxification method and have potential application in the treatment of diseases initiated by pore-forming toxins [76,77].

Recently, a similar method of toxin absorption by the erythroliposome (RM-PL) has been presented. In this method, the biomimetic core is made of artificial lipid membranes and erythrocyte membranes, which are supposed to neutralize various hemolytic pores-forming toxins. Tests performed with α-toxin, listeriolysin O, and streptolysin O showed that RM-PLs fully inhibit hemolysis in a concentration-dependent manner. In contrast, in vivo studies revealed that RM-PLs are effective in neutralizing various toxins and contribute to the survival of animals without damaging organs or tissues. Tested detoxification mechanisms showed that macrophages in the spleen and liver took the toxins absorbed into RM-PLs and digested them in lysosomes [78].

According to literary data, sphingomyelin and cholesterol contained in modified liposomes can be used as neutralizers of *S. aureus* and *Streptococcus pneumoniae* toxins [79]. Research showed that liposomes are five times more effective than clindamycin and can be particularly used in treatment of infections with bacteria living intracellularly [80].

Jabir et al. (2022) [81] found that Cur-Au@ZnO nanoparticles are highly effective as antibacterial agents and inhibitors of the Hla toxin produced by *S. aureus*.

It was also stated that Ag_3_PW_12_O_40_ nanoparticles (AgWPA-NPs) inhibit the growth of *S. aureus*, biofilm formation, expression of the SEA and SEB proteins, as well as disintegrate the cell membrane [82].

## 3. Antithrombotic Action of Used Compounds

Endothelial cells (ECs), which are highly important in hemostasis, are involved in blood flow and thrombosis. In vivo, the endothelium prevents thrombosis by triggering various anticoagulant and antiplatelet mechanisms. ECs regulate the clotting mechanism by controlling the expression of binding sites of anticoagulant and procoagulant factors on the cell surface. The endothelium protects against thrombosis through the transmission of tissue factor (TF) inhibitors and thrombin as well as receptors for the activation of protein [83,84].

Platelets are other important cells involved in hemostasis. Normally, platelets show adhesion, activation, and aggregation only after stimulation [85]. Upon adhesion, platelets undergo a release reaction in which substances such as ADP, serotonin, and thromboxane A2 (TxA2) can promote further aggregation by recruiting more platelets. Platelets can be activated in several ways. They contain receptors for agonists, such as arachidonic acid, collagen, thrombin, and prostaglandin endoperoxides, that can induce platelet aggregation. Leukocytes are also involved in formation of a thrombus in the venous vessel, together with ECs and platelets [86]. Endothelial activation leads to the adhesion of platelets and leukocytes. Under these conditions, leukocytes become activated and promote TF expression, activating the coagulation cascade. Moreover, the anticoagulant effect of the endothelial surface is suppressed by slow blood flow, leading to hypoxia and, in consequence, to the expression of endothelial adhesion molecules [87].

TF-induced activated clotting pathways are often present in sepsis patients. In addition, they decrease the levels of anticoagulants and fibrinolysis as well as activated endothelial surfaces and platelets. These unfavorable conditions contribute to formation of microclots in disseminated patients with intravascular coagulation, which leads to failure of many organs [88,89]. *S. aureus* virulence factors cause havoc in the cardiovascular system. These include proteins and exotoxins which contribute to thrombus formation by affecting the coagulation process and anticoagulation factors. PVL, for example, is responsible for damage to vascular endothelium and leukocyte lysis. Exotoxins also contribute to microthrombosis and deep venous thrombosis [11].

Table 1 presents the influence of different virulence factor groups on cell damage.

Studies have shown that the PVL damages neutrophils and, as a result, α-defensins, the product of myeloperoxidase, i.e., HOCl, and inhibit the formation of HOCl-modified proteins, which produce and activate platelets. Platelet activation inhibitors include α-defensin inhibitor, glutathione, and resveratrol—a plant polyphenol from the stilbene group [104].

Many compounds of plant origin are able to inhibit platelet activation and aggregation. Many of the abovementioned compounds are not only inhibitors of staphylococcal virulence factors but also have antiplatelet and/or anticoagulant properties.

Carvacrol can also inhibit the inflammatory cytokine levels; the expression of inducible nitric oxide synthase (iNOS) and cyclooxygenase-2 (COX-2); neutrophil elastase production; and the production of prostaglandins E2 (PGE2), prostaglandins F1 (PGF1), and prostaglandins F2 (PGF2) [105].

El Azab et al. (2022) [106] proved that geraniol displayed an effect of reducing the expression and release of cellular proinflammatory cytokines, tumor necrosis factor-α (TNF-α), IL-1β, IL-8, and nitric oxide, an upregulating the gene expression of anti-inflammatory cytokine IL-10 in lipopolysaccharide, and inducing white blood cells. They assessed its anti-inflammatory effects as superior to those of diclofenac. Research shows that its anti-inflammatory and antithrombotic effects are due to a good affinity toward apoptosis signal-regulating kinase 1 (ASK1) and human P2Y12 receptors.

Flavonoids constitute the largest group of antiplatelet and/or anticoagulant factors; other compounds include anthraquinone derivatives and xanthon. These factors are important in the event of staphylococcal infections, as they can lead to intravascular coagulation and damage to host organs. The presented compounds would, on the one hand, prevent development of infection and, on the other hand, would protect the host against thrombosis. It is known that the activation of platelets is important for hemostasis signaling but also in atherosclerosis and thrombosis [107,108].

Resveratrol shows the ability to inhibit platelet aggregation and an anticoagulant effect in vivo. It was shown that thrombin-induced transport of calcium ions to platelets, and consequently platelet aggregation, is significantly inhibited by resveratrol. Resveratrol inhibits thrombin-induced platelet aggregation by inhibiting the release of Ca^2+^ from its stores and inhibiting the influx of Ca^2+^ from the store to the platelets. However, its low bioavailability and fast metabolism make it less effective [109]. Resveratrol suppresses macrophages/mast cell-derived proinflammatory factors, including PAF (platelet-activating factor), TNF-α, and histamine. Administration of resveratrol as an antioxidant may contribute to a better prognosis in stroke treatment [110].

It has been recently shown that naringenin, depending on the dose, inhibits agonist-induced platelet aggregation in in vitro conditions and also stimulated by ADP. This flavonoid also inhibits ex vivo ADP-initiated platelet aggregation. Naringenin inhibits the release of ADP-stimulated α-granules of platelets and the binding of fibrinogen and slows down the expansion of platelets into fibrinogen and intracellular mobilization of calcium ions. It prevents blocking of the adhesion of platelets to the surface covered with collagen. Its action involves an attenuation of PI3K-signaling and platelet phosphodiesterase activities. It was also found that naringenin increases the cGMP level and VASP phosphorylation at Ser239, whereas the PKA inhibitor causes dephosphorylation of VASP and eliminates platelet aggregation initiated by this flavonoid. It also appears that naringenin may be a potential antiplatelet compound involved in PI3K and cyclic nucleotide signaling and is characterized by a low risk of bleeding. The basis for such conclusions is provided by studies conducted on rats, in which inhibition of clot formation was observed in the carotid thrombus model and studies on mice revealed that naringenin did not prolong the bleeding period [111].

Earlier reports have shown that some flavonoids inhibit platelet function through various mechanisms, including blockade of TxA2 receptors (TPs). Binding tests showed that apigenin is an effective binding competitor to aspirin, and perfusion studies showed that flavonoids, apigenin, genistein, and catechin significantly reduce thrombus formation. Apigenin significantly extended the closure time of PFA-100 induced by collagen and epinephrine. When added to platelets, previously exposed in vivo to aspirin, it enhanced its inhibitory effect on platelet aggregation. The inhibitory effect of some flavonoids in the presence of plasma, mainly apigenin, may partly depend on the antagonism of the TP. An increased antiplatelet effect of aspirin in the presence of apigenin ex vivo encourages a combined administration of aspirin from some flavonoids in patients in whom aspirin is insufficient to suppress the TxA_2_ pathway [112].

Garcinia species contain many valuable bioactive compounds with therapeutic properties, including anti-inflammatory, antiplatelet, antioxidant, antibacterial, analgesic, and other properties [113]. Garcinia species (*Clusiaceae*) include compounds, mainly polyisoprenylated benzophenones, flavonoids, and xanthones [114]. Among many Garcinia plant extracts, *Garcinia eugenifolia* wall leaf extract shows the highest activity in inhibiting LDL peroxidation, and *Garcinia mangostana Linn*. extract shows the most effective activity against platelet aggregation initiated by arachidonic acid and collagen [115].

The anti-aggregation activity of hydroxytyrosol (HT), monophenol, and its acetate ester hydroxytyrosol acetate (HT-AC) in human whole blood was investigated and compared with acetylsalicylic acid (ASA). HT-AC and HT were shown to inhibit ADP, collagen, or arachidonic-acid-initiated platelet aggregation in both whole blood and platelet-rich plasma. All tested compounds inhibited the production of thromboxane B2 in platelets and 6-keto-prostaglandin F1alpha (6-keto-PF1alpha) in leukocytes and also stimulated the production of nitric oxide. On the other hand, higher concentrations of about 500 μM (HT) suppressed the production of 3-nitrotyrosine. In addition, HT, HT-AC, and ASA inhibited the production of TNF-α by leukocytes [116]. Another study showed that HT stimulates the PI3K/AKT/mTOR pathways and inhibits inflammatory factors and mediators such as IL-1β, IL-6, E-selectin, P-selectin, VCAM-1, and ICAM-1 in endothelial vascularization and functioning. It was also observed that HT impairs the vascular smooth muscle (SMC) of the cell cycle by dephosphorylation of ERK1/2 and AKT pathways. Moreover, HT was shown to promote the endothelium and inhibit vascular SMC migration, thereby inhibiting the development of intimal hyperplasia [117].

**Figure 7:**
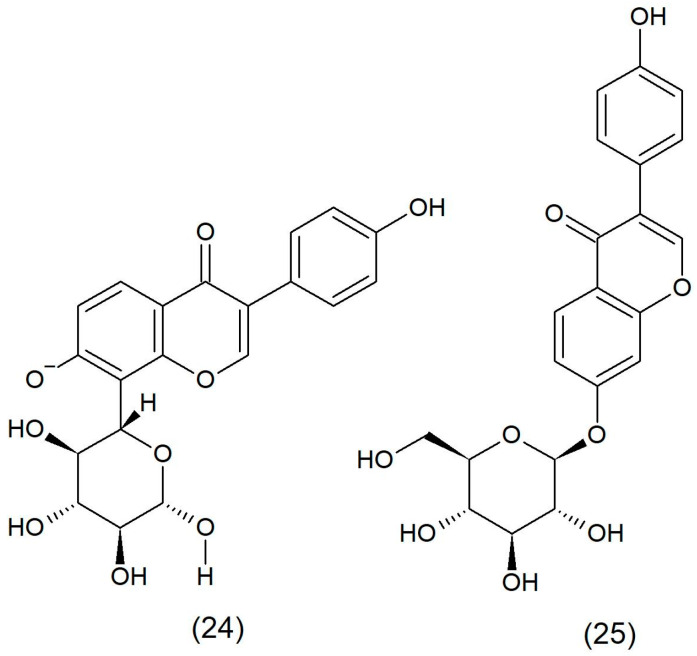
Chemical structure of puerarin (**24**) and daidzin (**25**).

Puerarin (Figure 7) and daidzin (Figure 7), isolated from the *Pueraria lobata rhizome*, inhibited ADP and collagen-induced platelet aggregation. However, daidzein showed a stronger inhibitory effect. Both compounds also showed an antiallergic effect. In vivo, puerarin and daidzin showed anticoagulant activity against collagen and epinephrine and prevented mice from dying from pulmonary thrombosis. Puerarin and daidzin are prodrugs with antiallergic and anticoagulant activity produced by intestinal microflora [118].

Oroxylin A prolonged the activated partial thromboplastin time (aPTT) and prothrombin time (PT) and inhibited the thrombin and activated factor X (FXa) activity in human umbilical vein endothelial cells (HUVECs). Oroxylin A (OroA) inhibited thrombin-catalyzed fibrin polymerization and platelet aggregation. OroA was also an anticoagulant in mice. OroA inhibited the production of TNF-α-induced plasminogen activator inhibitor type 1 (PAI-1). Treatment with OroA led to a significant decrease in the PAI-1-to-t-PA ratio (tissue plasminogen activator). These results clearly show the anticoagulant effect of oroxylin A as a potential antithrombotic agent [119].

Chelerythrine is a well-known protein kinase C (PKC) inhibitor. It can also act on cytoskeleton proteins, which are associated with deformability of RBCs [120].

Much evidence indicates that histone deacetylases (HDACs) play an important role in the development of atherosclerosis. HDACs can regulate most of risk factors associated with atherosclerosis [121]. One of HDAC inhibitors is apicidin, which normalizes the production of tissue-type plasminogen activator (t-PA) and is a modulator of endogenous fibrinolysis [122].

## 4. Conclusions and Perspectives

The announcement of the post-antibiotic era has increased the scientific activity in the development of strategies for the treatment of bacterial infections, including those caused by multidrug-resistant staphylococci. It has long been known that these bacteria have a rich arsenal of virulence factors, including those responsible for thrombosis. The presented review shows that compounds of various origins may have the ability to neutralize, interfere with, and inhibit the expression of staphylococcal toxins. Due to their antithrombotic properties, they can prevent thromboembolic complications caused by infection.

The results of preliminary studies show that flavonoids and other compounds discussed in this work can be used in the treatment or prevention of infections through infection prophylaxis. Therefore, the use of an appropriate diet with the participation of bioactive substances has a health-promoting effect, including antibacterial effects. Flavanoids and other substances of plant origin exhibit a wide range of diverse effects, including inhibition of cell wall synthesis, the ability to disrupt the integration of cell membranes, influence on the formation and development of bacterial biofilms, limitation of their adhesion, disturbances in the synthesis of cell envelopes, influence on the synthesis and structure of nucleic acids, inhibition of bacterial toxins, and influence on the electron transport chain and ATP synthesis.

Natural substances as an antimicrobial tool exhibit many advantages, such as availability, different structural structure, and strong bioactivity, and are characterized by a variety of mechanisms of action. Importantly, the latest in vivo studies have confirmed their beneficial effects in animal models in reducing bacterial infections and improving the host’s condition without side effects.

In turn, nanosponges can be used both to absorb bacterial toxins and to transport drugs/natural substances directed against pathogens.

Further studies on the use of monoclonal antibodies for a better diagnosis and potential treatment of biofilm-related infections, insensitive to antibiotics, for example, in endocarditis, prosthetic joints, catheters, and others, will also be of great importance.

Despite promising results, comprehensive clinical and long-term studies are necessary to assess the safety, the most favorable dosage, and the efficacy in humans. Studies are also needed to precisely explain the mechanisms at the molecular level underlying their antibacterial activity. Additionally, it would be necessary to search for more potent derivatives/analogs of proven compounds and optimize their use in combination therapies.

## Figures and Tables

**Table 1:** The virulence factor groups and their influence on cell damage.

Virulence Factors	Role of Virulence Factors on Host Cell’s	References
Enterotoxins	Immune cell activation by superantigens. Activation of ECs and T cells by SEB. The interaction of enterotoxins with epithelial cells: disruption of the integrity of cell membranes increasing intestinal permeability 4. ECs damage: cytotoxic effects—binding superantigens to luminal surface of ECs T cell-mediated lysis of ECs 5. Activation of a large number of T cells: intensify immune response production of inflammatory mediators development of inflammation 6. Activation of fibroblasts, epithelial and ECs by IL-1 and TNF-α to produce other mediators for T-cell activation. 7. Stimulation of non-specific T-cell proliferation by superantigens.	[90,91,92,93]
Adhesins	Various host ligands in vessel walls can be a tie point. Fibrinogen is binding by *S. aureus* surface adhesins (ClfA, ClfB). Von Willebrand factor (vWF) on activated endothelium is binding by staphylococcal vWF-binding protein (vWbp). Collagen in damage endothelial layer binds either directly with the collagen adhesin (Cna), or indirectly with vWF. Fibronectin-binding proteins A and B (FnBPA and FnBPB) can mediate in adhesion to and internalization of *S. aureus* in ECs. IsdB binds to integrin αvβ3 on ECs and to integrin GPIIb/IIIa on platelets—promotes adherence and internalization. IsdB acts as a receptor for vitronectin, whitch mediates adherence to HUVEC. Surface adhesins to clump in blood and adhere to vessel walls, leading to endothelial damage, and secondary infectious foci. Specific binding of MSCRAMMs to fibronectin, fibrinogen, and collagen.	[94,95,96,97]
Exotoxins	The binding of α-toxin to its receptor A-disintegrin and ADAM10 damage the endothelial barrier function. β-toxin causes a decrease the pro-angiogenic molecule MMP-8 and increase the anti-angiogenic molecule endostatin. β-toxin induces vascular leakage, neutrophilic inflammation, decreases expression of the IL-8 and upregulates expression of VCAM-1. β-toxin targets human EC proliferation. The leukocidins cause pores in the cell membrane, which results in disintegration of leukocytes and RBCs. LukED and HlgAB cause vascular congestion and disorders in vascular fluid flow. LukED and HlgAB injure primary human endothelial cells in a DARC-dependent manner. PVL lysis neutrophils and release ROS, which consequently leads to pulmonary vascular damage. Phenol-soluble modulin (PSMα) lysis neutrophils, RBCs, and it cause induce vascular leakage.	[98,99,100,101]
Exoenzymes	Coagulases and staphylokinases control the host’s coagulation system. Nucleases and proteases cleave and inactivate complement factors, surface receptors, and AMPs. Some exoenzymes can cause disruption of epithelial and endothelial barriers through by influencing the structure of proteins responsible for, among others, vascular wall permeability, thrombotic and inflammatory processes, and angiogenesis.	[102,103]

## Data Availability

No new data were created or analyzed in this study.

## Abbreviations

The following abbreviations are used in this manuscript:

6-ES	ethoxysanguinarine
6-MS	6-methoxydihydrosanguinarine
ADP	adenosine diphosphate
AI-2	autoinducer-2
AMP	antimicrobial peptide
aPTT	activated partial thromboplastin time
ASA	acetylsalicylic acid
ASK1	apoptosis signal-regulating kinase 1
ATP	adenosine triphosphate
CH	chelerythrine
Cna	collagen adhesin
COX-2	cyclooxygenase-2
DICH	dihydrochelerythrine
DNA	deoxyribonucleic acid
EC	endothelial cell
FDA	Food and Drug Administration
FnBPA	fibronectin-binding protein A
FnBPB	fibronectin-binding protein B
HBD-1	human β-defensin 1
HD	human α-defensin
HDAC	histone deacetylase
Hla	α-hemolysin
HNP	human neutrophil peptide
HT	hydroxytyrosol
HT-AC	hydroxytyrosol acetate
HUVEC	human umbilical vein endothelial cell
iNOS	inducible nitric oxide synthase
IsdB	iron-regulated surface determinant protein B
IVIg	intravenous immunoglobulin
LDL	low-density lipoprotein
LukAB	leukocidin AB
MDR	multidrug-resistant
MIC	minimal inhibitory concentration
MRSA	methicillin-resistant *Staphylococcus aureus*
MSCRAMMs	microbial surface components recognizing adhesive matrix molecules
MSSA	meticillin-sensitive *Staphylococcus aureus*
NP	nanoparticle
OroA	oroxylin A
PAI-1	plasminogen activator inhibitor type 1
PGE2	prostaglandin E2
PGF1	prostaglandin F1
PGF2	prostaglandin F2
PIA	polysaccharide intercellular adhesin
PKC	protein kinase C
PLGA	polylactic-co-glycolic acid
PSMα	phenol-soluble modulin
PT	prothrombin time
PVL	Panton-Valentine leukocidin
QS	quorum sensing
rAT	recombinant α-toxoid
RBC	red blood cell
RM-PL	erythroliposome
RNA	ribonucleic acid
ROS	reactive oxygen species
rTSST-1v	toxic shock syndrome toxin 1 variant vaccine
SE	staphylococcal enterotoxin
SEA	staphylococcal enterotoxin A
SEB	staphylococcal enterotoxin B
SMC	vascular smooth muscle cell
SplB	*Staphylococcus aureus*-serine protease-like protein B
SrtA	sortase A
SSTIs	skin and soft tissue infections
TF	tissue factor
TNF-α	tumor necrosis factor-α
TP	TxA2 receptor
t-PA	tissue-type plasminogen activator
TSS	toxic shock syndrome
TSST-1	toxic shock syndrome toxin-1
TxA2	thromboxane A2
vWbp	vWF-binding protein
vWF	von Willebrand factor
